# The Proteasome Inhibitor Bortezomib Sensitizes AML with Myelomonocytic Differentiation to TRAIL Mediated Apoptosis

**DOI:** 10.3390/cancers3011329

**Published:** 2011-03-15

**Authors:** Marianne van Dijk, Eoin Murphy, Ruth Morrell, Steven Knapper, Michael O'Dwyer, Afshin Samali, Eva Szegezdi

**Affiliations:** 1 Apoptosis Research Center, National University of Ireland, University Road, Galway, Ireland; E-Mails: m.vandijk1@nuigalway.ie (M.V.D); Eoin.murphy@nuigalway.ie (E.M); ruth.morrell@nuigalway.ie (R.M); afshin.samali@nuigalway.ie (A.S); michael.odwyer@hse.ie (M.O.); 2 School of Natural Sciences, National University of Ireland, University Road, Galway, Ireland; 3 School of Medicine, National University of Ireland, University Road, Galway, Ireland; 4 Department of Haematology, School of Medicine, Cardiff University, Heath Park, CF14 4XN Cardiff, UK; E-Mail: KnapperS@cf.ac.uk

**Keywords:** AML, FAB M4/M5, TRAIL, bortezomib, apoptosis, NF-κB, c-FLIP

## Abstract

Acute myeloid leukemia (AML) is an aggressive stem cell malignancy that is difficult to treat. There are limitations to the current treatment regimes especially after disease relapse, and therefore new therapeutic agents are urgently required which can overcome drug resistance whilst avoiding unnecessary toxicity. Among newer targeted agents, both tumor necrosis factor (TNF)-related apoptosis-inducing ligand (TRAIL) and proteasome inhibitors show particular promise. In this report we show that a combination of the proteasome inhibitor bortezomib and TRAIL is effective against AML cell lines, in particular, AML cell lines displaying myelomonocytic/monocytic phenotype (M4/M5 AML based on FAB classification), which account for 20-30% of AML cases. We show that the underlying mechanism of sensitization is at least in part due to bortezomib mediated downregulation of c-FLIP and XIAP, which is likely to be regulated by NF-κB. Blockage of NF-κB activation with BMS-345541 equally sensitized myelomonocytic AML cell lines and primary AML blasts to TRAIL.

## 1. Introduction

Acute myeloid leukemia (AML) is an aggressive stem cell malignancy characterized by proliferation and accumulation of immature hematopoietic cells in the bone marrow and peripheral blood, leading to the clinical manifestations of bone marrow failure. With standard anthracycline and cytarabine based induction chemotherapy, complete remission (CR) is achieved in 50–80% of patients considered suitable for intensive treatment [1]. However, despite achievement of CR and subsequent post-remission therapy, the majority of patients relapse and die of their disease [1]. Moreover, many older patients are not deemed suitable for intensive therapy due to excessive treatment related mortality. The optimal treatment for these older patients has not been defined. Thus, overall five-year survival in adults remains less than 50% in patients under 45 years of age and <5% in patients over 65 years at diagnosis [2]. The poor survival in older patients is particularly important given that the majority of patients belong to this age group.

Relapse of AML following chemotherapy is likely due to the persistence of resistant leukemic cells localized within the bone marrow [2]. This resistance may be attributed to intrinsic properties of the leukemic stem or progenitor cells, as well as to their interaction with the bone marrow microenvironment. Due to the limitations of current regimes, new chemotherapeutic agents are urgently required, which can overcome drug resistance whilst avoiding unnecessary toxicity. Among the newer targeted agents, both tumor necrosis factor (TNF)-related apoptosis-inducing ligand (TRAIL) and proteasome inhibitors show particular promise [3].

A major challenge in the treatment of all malignancies remains the destruction of cancer cells whilst sparing normal cells. TRAIL is a type II transmembrane protein with the ability to promote apoptosis, in a p53-independent manner, by engaging the death receptors DR4 and DR5 while sparing most normal cells [4]. It is active in a variety of tumor cell lines and xenotransplant mouse models thus making it a promising anti-cancer cytokine [5-7]. However, many primary tumors, including AML, are resistant to the pro-apoptotic effects of recombinant human TRAIL (rhTRAIL) through a variety of mechanisms including reduced expression of DR4/DR5, elevated expression of TRAIL decoy receptors [8] and increased expression of anti-apoptotic proteins such as cellular-FLICE-inhibitory protein (c-FLIP) and X-linked inhibitor of apoptosis protein (XIAP) [9-11]. As a consequence, stimulation of the death receptors results in alternative signaling with activation and nuclear translocation of nuclear factor-κB (NF-κB), which, in turn, leads to the induction of anti-apoptotic and prosurvival genes further increasing resistance [12].

In an attempt to bypass this resistance, various anti-tumor agents have been combined with rhTRAIL to study their sensitizing effects. Of increasing interest is the combination of rhTRAIL with proteasome inhibitors, such as bortezomib, a highly selective, reversible inhibitor of the chymotrypsin-like activity of the proteasome complex [13-15]. The 26S proteasome is a large intracellular multisubunit protease (1500–2000 kDa) that consists of one 20S catalytic core complex associated with 19S or 11S regulatory complexes. It has three distinct catalytic properties, namely a trypsin-like, a chymotrypsin-like and a peptidyl-glutamyl peptide hydrolyzing activities [16-18]. The ubiquitin–proteasome system (UPS) mediates the degradation of polyubiquitinated proteins and represents one of the main pathways of protein degradation in eukaryotic cells. The pronounced apoptosis-inducing activity of bortezomib has been demonstrated in many types of cancer [19,20]. Proposed mechanisms include (i) intracellular accumulation of p53, the IκB/NF-κB complex leading to NF-κB inhibition and pro-apoptotic members of the Bcl-2 family [19,21] and (ii) altered cell cycle regulation through stabilization of cyclins and the CDK inhibitors p21 and p27 [22].

AML cells display varying *in vitro* sensitivity to apoptosis induced by proteasome inhibitors [8,23]. Studies have shown that human leukemic cells express abnormally high levels of proteasomes, compared with their normal counterparts [24] and that activity patterns of the various subunits varies in primary leukemia cells, which reflects their sensitivity to bortezomib [25]. In addition, many cases of AML show increased activation of the survival-signaling pathway mediated by NF-κB [26], particularly the leukemic stem cell component, and importantly, it has been shown that leukemic stem cells are more susceptible to bortezomib-induced apoptosis than their normal counterparts [27]. In the clinical setting, however, bortezomib has limited single agent activity possibly due to its relatively low maximum tolerated dose [28]. Combination approaches have higher potential with a promising CR rate being achieved in a high-risk cohort of patients when bortezomib was combined with standard AML induction therapy [29]. However, as most elderly patients with AML do not tolerate intensive induction chemotherapy, investigation of other novel combinations is needed.

The combination of rhTRAIL and bortezomib has shown synergistic apoptotic responses in a variety of malignancies including chronic lymphocytic leukemia and AML [8,30], non-small cell lung cancer [31] and non-Hodgkin's lymphoma [32]. Although the precise molecular mechanisms are unclear, in many instances the stimulatory effects of bortezomib on TRAIL receptor DR4/DR5 expression and the downmodulation of c-FLIP seem to be key mediators in the enhancement process [33], although this may be cell-type specific. Sensitised tumor cells may also demonstrate stronger DISC formation, with increased FADD and caspase-8 recruitment [34]. Finally, blockage of TRAIL-induced NF-κB activation by bortezomib may also play a role [32].

In the present work, we explored the sensitivity of several AML cell lines to rhTRAIL and the proteasome inhibitor, bortezomib. In addition, we evaluated the ability of bortezomib to co-operate with rhTRAIL as well as exploring the mechanism underlying their co-operation. The results indicate that, particularly in cell lines of myelomonocytic differentiation, pre-treatment with bortezomib enhances rhTRAIL-induced apoptotic signaling both in rhTRAIL-sensitive and TRAIL-resistant cell lines. We demonstrate that the inhibition of NF-κB activation plays a central role in sensitizing AML to rhTRAIL-induced apoptosis. In addition, treatment with bortezomib caused a downregulation of c-FLIP and in specific cell lines, XIAP, further strengthening the importance of NF-κB inhibition as a molecular basis to explain sensitization of myelomonocytic AML cells to rhTRAIL.

## 2. Results and Discussion

### 2.1. AML Cell Lines Show Varied Resistance to TRAIL

To investigate the effect of rhTRAIL on the growth and survival of AML cells, a panel of AML cell lines was chosen representing different stages of differentiation. KG-1 cells are phenotypically characterized as immature or minimally differentiated-FAB (French-American-British) classification M0, Kasumi as FAB M2, HL-60 as promyelocytic (M3) and the remaining cell lines ML-1, ML-2, OCI AML2, OCI AML3 as myelomonocytic (M4) or monocytic (M5a: MOLM13). Cells were treated with increasing concentrations of rhTRAIL (10 to 1000 ng/mL) for 24 h. Following treatment, cell viability was measured by MTT assay.

**Figure 1.**
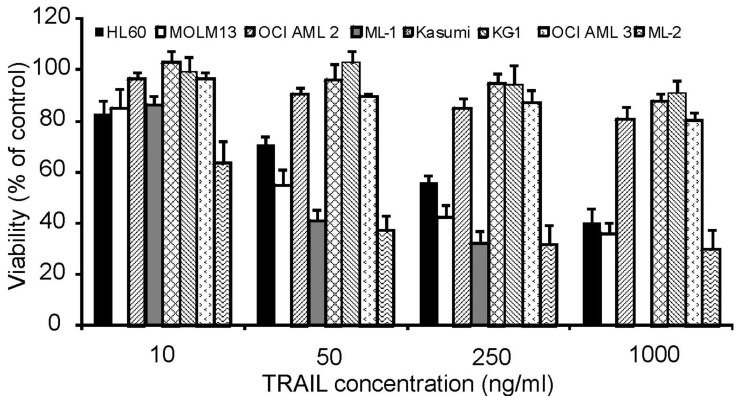
Acute myeloid leukemia (AML) cell lines show varied resistance to rhTRAIL. AML cell lines were treated with 10, 50, 250 or 1000 ng/mL TRAIL for 24 h. Cell viability was measured by MTT assay; values are expressed as a percent of untreated cells and presented as mean ± S.E.M.

The cell lines exhibited variable sensitivities to rhTRAIL. Kasumi, KG-1, OCI AML2 and OCI AML3 were highly resistant showing a cell viability of >80% at the highest rhTRAIL concentration, while HL-60, ML-1, ML-2 and MOLM13 cells were more susceptible to the cytotoxic effects of rhTRAIL in a dose dependent manner (Figure 1).

### 2.2. AML Cell Lines are Highly Sensitive to Bortezomib

**Figure 2.**
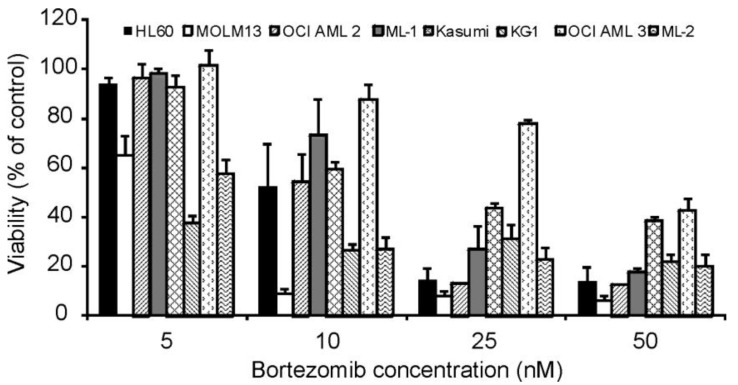
AML cell lines are highly sensitive to bortezomib. AML cell lines were treated with 5, 10, 25 or 50 nM bortezomib for 24 h after which cell viability was measured by MTT assay. Values are expressed as a percent of untreated cells and presented as mean ± S.E.M.

Bortezomib is a boronic acid dipeptide, which acts as a reversible inhibitor of the 26S proteasome. The panel of AML cell lines were treated with increasing concentrations of bortezomib for 24 h after which cell viability was measured by MTT assay. All cell lines were sensitive to bortezomib; however, the degree of sensitivity varied (Figure 2) with OCI AML3 being the most resistant, requiring a dose of >25 nM bortezomib to show a 50% reduction in cell viability (Figure 2). KG-1 and MOLM13 cells exhibited the greatest sensitivity with a reduction in cell viability of approximately 62% and 37%, respectively, following treatment with 5 nM of bortezomib. In all cases, the effect on cell viability was dose-dependent and reached a plateau.

### 2.3. Bortezomib Treatment Inhibits the Chymotryptic Activity of the Proteasome in AML Cell Lines

**Figure 3.**
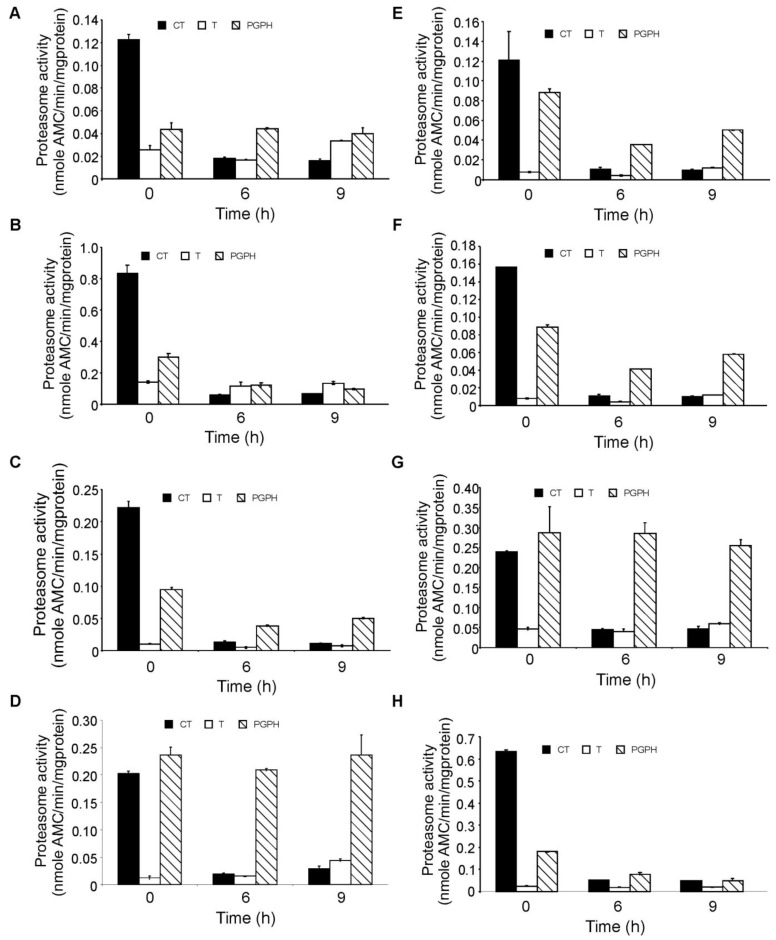
Bortezomib inhibits the chymotryptic activity of the proteasome. AML cells were treated with 100 nM bortezomib for 6, 9 or 18 h. Chymotryptic-like (CT), tryptic-like (T) and PGPH-like proteasomal activity was measured as described in the experimental section Proteasome activity is expressed as nmoles substrate (AMC) cleaved per 1 mg cellular protein in one minute ± S.E.M. (**A**) HL-60; (**B**) KG-1; (**C**) Kasumi; (**D**) OCI AML2; (**E**) OCI AML3; (**F**) MOLM13; (**G**) ML-2; (**H**) ML-1.

In order to investigate the degree of inhibition of the proteasome following bortezomib treatment, the three different enzymatic activities of the proteasome, chymotrypsin-like, trypsin-like and peptidyl-glutamyl-peptidyl hydrolase (PGPH)), were measured in the panel of AML cell lines after 6 and 9 h of exposure. Using a concentration of 100 nM (previously shown to induce almost complete cell death in all cell lines) bortezomib almost completely inhibited the chymotryptic activity of the proteasome in all cell lines cells after 6 h of treatment (Figure 3). Tryptic activity was the lowest of the three enzymatic activities and remained unaffected by bortezomib treatment in all cell lines while the PGPH activity was partially inhibited apart from in HL-60, ML-2 and OCI AML2 cells in which PGPH activity was unaffected by bortezomib, however these cell lines did not show higher or lower sensitivity to bortezomib.

### 2.4. Degree of Proteasome Inhibition does not Correlate with Bortezomib Sensitivity

**Figure 4.**
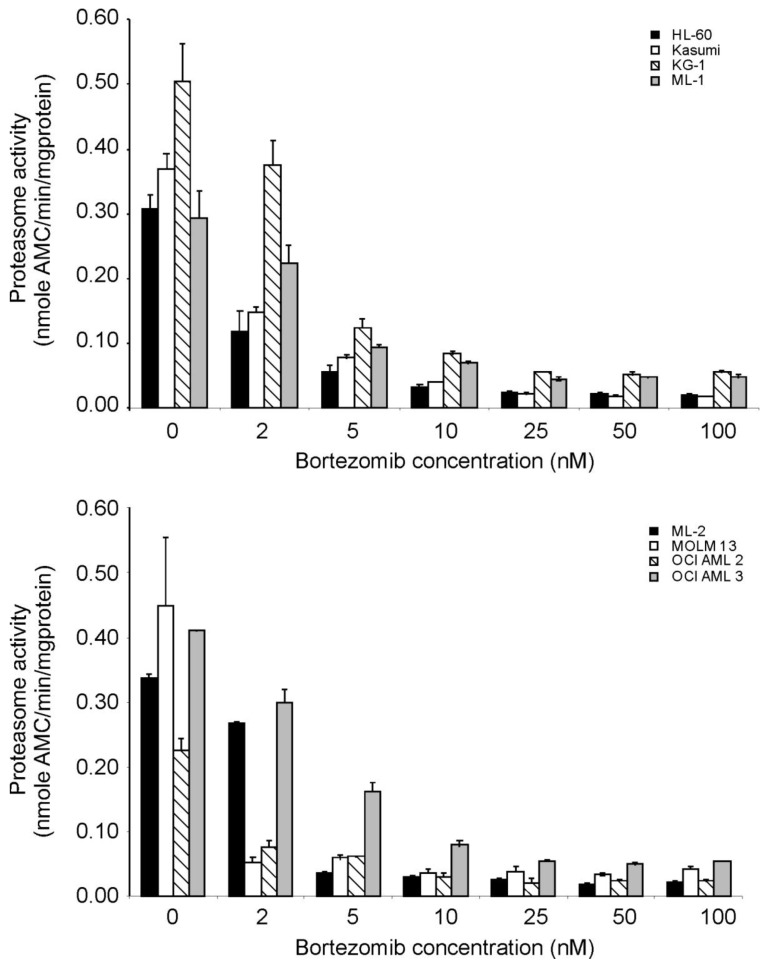
Proteasome inhibition increases with increasing concentration of bortezomib. AML cells were treated with increasing doses of bortezomib (0–100 nM) for 6 h. Proteasome activity is expressed as nmoles substrate (AMC) cleaved per 1 mg cellular protein in one minute ± S.E.M.

To further study the effect of bortezomib on the chymotryptic activity in AML cell lines, the panel of AML cells were treated with a range of bortezomib concentrations (2 nM to 100 nM) for 6 h and the chymotryptic proteasomal activity was measured. The highest basal chymotryptic activity was seen in the MOLM13 and KG-1 cell lines, the two cell lines with the highest bortezomib sensitivity. The cell lines were grouped according to the degree of proteasomal inhibition obtained, with MOLM13, OCI AML2, HL60 and Kasumi cells being the most sensitive (IC_50_ value of 1.9 ± 0.5 nM), while OCI AML3, KG-1, ML-1 and ML-2 cells were more resistant (IC_50_ value of 5.1 ± 0.4 nM) requiring approximately 2.5-fold more bortezomib for half-maximal inhibition of the chymotryptic activity of the proteasome (Figure 4). Overall, in all cell lines, a low nanomolar concentration of bortezomib was sufficient to reduce proteasome activity to less than 20% of the basal activity. However, the sensitivity to proteasomal inhibition does not correlate tightly with the effects of bortezomib on cell viability (Figure 2). For example, KG-1 and MOLM13 are both sensitive to bortezomib in cell viability assays but chymotryptic activity assays show that chymotryptic activity is inhibited at low concentrations of bortezomib in MOLM13, whereas higher concentrations of bortezomib are required to inhibit chymotryptic activity in KG-1.

### 2.5. Bortezomib Sensitizes Myelomonocytic AML Cells to TRAIL-Induced Apoptosis

**Figure 5.**
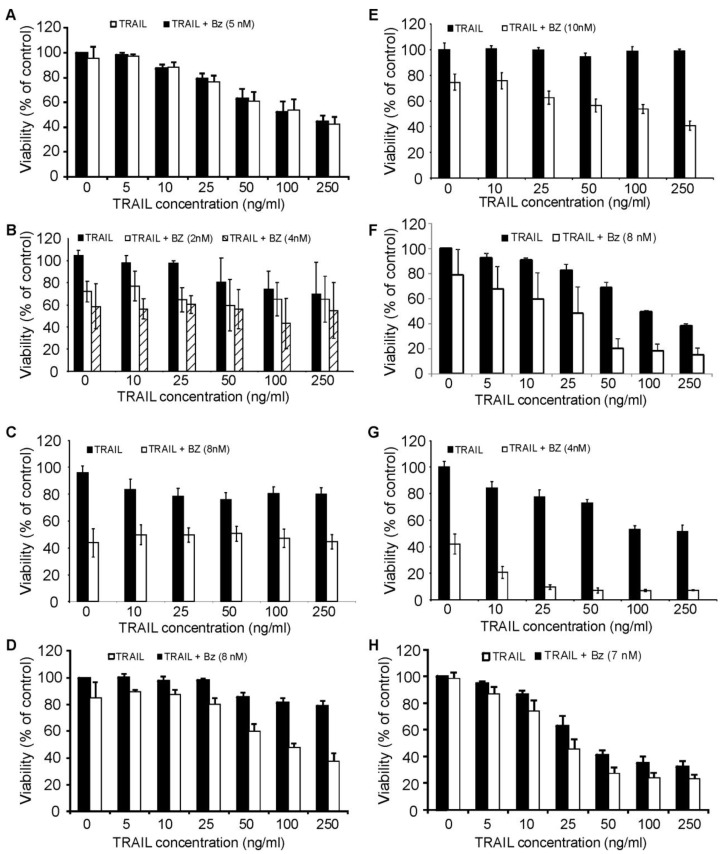
Bortezomib sensitizes ML-1, ML-2, OCI AML2, OCI AML3 and MOLM13 to TRAIL induced apoptosis. AML cells were pre-treated with a sublethal dose of bortezomib that induced 20% cell death (2 nM (KG-1), 4 nM (KG-1, ML-2), 5 nM (HL-60), 7 nM (ML-1), 8 nM (Kasumi, OCI AML2, MOLM13) or 10 nM (OCI AML3)) for 18 h followed by a 18 h (KG-1, Kasumi, OCI AML3, ML-2) or 24 h (HL-60, ML-1, OCI AML2, MOLM13) treatment with 5, 10, 25, 50, 100, 250, 500 or 1000 ng/mL TRAIL. Cell viability was measured by MTT assay; values are expressed as a percent of untreated cells and presented as mean ± S.E.M. (**A**) HL-60; (**B**) KG-1; (**C**) Kasumi; (**D**) OCI AML2; (**E**) OCI AML3; (**F**) MOLM13; (**G**) ML-2; (**H**) ML-1.

We next evaluated the effect of bortezomib-induced proteasome inhibition in combination with rhTRAIL-mediated death in the panel of AML cell lines. Cells were treated with a concentration of bortezomib that induced approximately 20% reduction in viability and was sufficient to inhibit the chymotrypsin-like activity of the proteasome. Pre-treatment of cells with bortezomib for 18 h followed by rhTRAIL treatment for a further 18 h or 24 h led to TRAIL sensitization in ML-1, ML-2, OCI AML2, OCI AML3 cells and MOLM13 (Figure 5D, 5E, 5F, 5G, 5H). Interestingly, all of these cell lines are of either myelomonocytic (M4) or monocytic (M5) differentiation. The synergistic action of the two agents was confirmed with a combination index (CI) <1 using the Chou-Talalay method (Table 1). Pretreatment with bortezomib did not sensitize HL-60, KG-1 and Kasumi to rhTRAIL-induced apoptosis (Figure 5A, 5B and 5C). Results were confirmed using Annexin V/PI assay (data not shown).

### 2.6. Molecular Mechanism of Bortezomib-Induced Sensitization of Tumor Cells to rhTRAIL

Roué and colleagues have shown that TRAIL resistance in mantle cell lymphoma cells is linked to NF-κB regulated expression of c-FLIP, the main endogenous regulator of caspase-8 activation [35]. Expression of XIAP, another important inhibitor of TRAIL-induced apoptosis, has also been shown to be NF-κB-dependent [36].

**Figure 6.**
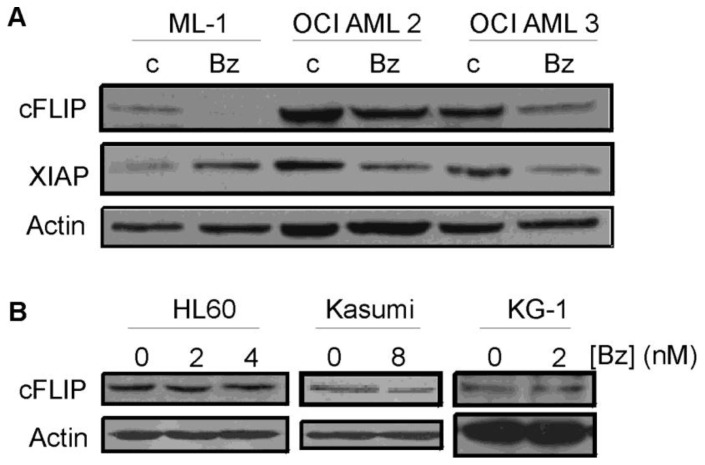
Bortezomib treatment leads to downregulation of c-FLIP and XIAP in FAB M4/M5 AML cells. Cells were treated with (**A**) 7 nM (ML-1), 16 nM (OCI AML2) or 25 nM (OCI AML3) bortezomib or (**B**) 2 nM (HL60, KG-1), 4 nM (HL60) or 8 nM (Kasumi) bortezomib for 18 h after which cell lysates were harvested and analyzed for c-FLIP and XIAP expression by Western blotting. Expression of actin was detected to serve as a loading control.

Since bortezomib can potentially inhibit NF-κB activation, we analyzed the effect of bortezomib on the relative expression of the anti-apoptotic proteins, c-FLIP and XIAP in ML-1, OCI AML2 and OCI AML3 cells. The expression of c-FLIP was downregulated after bortezomib treatment (Figure 6A) in all three cell lines. XIAP was also downregulated post treatment with bortezomib with the exception of ML-1 cells which exhibited an increased expression, an effect which may be mediated by inhibition of XIAP degradation via the proteasome. In addition, we analyzed the effect of bortezomib on the expression of c-FLIP in HL-60, Kasumi and KG-1 cells. On the contrary, bortezomib did not reduce the expression of c-FLIP in HL-60, Kasumi and KG-1 cells, cell lines which bortezomib could not sensitize for TRAIL-induced apoptosis (Figure 6B).

**Figure 7.**
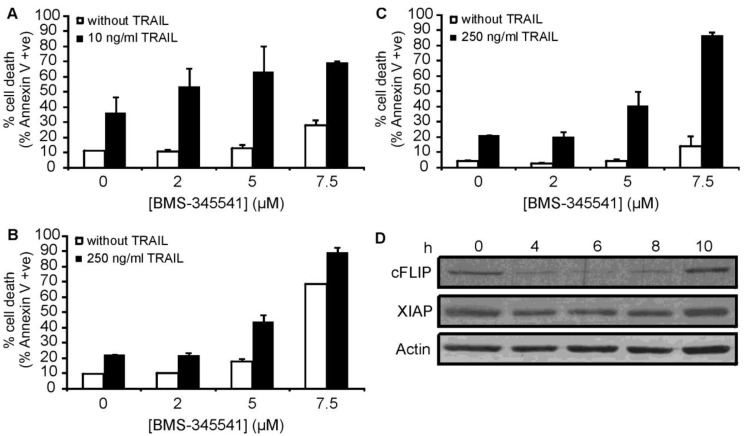
NF-κB inhibition sensitizes ML-1, OCI AML2 and OCI AML3 to TRAIL-induced apoptosis. (**A**-**C**) AML cells were pre-treated with 2, 5 or 7.5 μM BMS-345541 for 15 h followed by a treatment with 10 ng/mL (ML-1) or 250 ng/mL TRAIL (OCI AML2 and OCI AML3) for 24 h. Induction of cell death was measured by Annexin V staining. Data shown are mean ± S.E.M. (**A**) ML-1; (**B**) OCI AML2; (**C**) OCI AML3; (**D**) OCI AML2 cells were treated with 5 μM BMS-345541 for 0, 4, 6, 8 or 10 h after which cell lysates were harvested and analyzed for c-FLIP and XIAP expression by Western blotting. Expression of actin was detected to serve as a loading control.

As both c-FLIP and XIAP are known to be regulated by the NF-κB pathway, our data supports the theory that inhibition of NF-κB activation plays a central role in the sensitization of these particular AML types to TRAIL-induced apoptosis. To further investigate the role of NF-κB in bortezomib-mediated sensitisation, ML-1, OCI AML2 and OCI AML3 cells were pre-treated with the inhibitor-κB kinase (IKK) inhibitor BMS-345541 [37] for 15 h followed by a 24 h treatment with rhTRAIL. BMS-345541 could sensitize all three cell lines to rhTRAIL-induced apoptosis (Figure 7A-C), which was associated with the downregulation of c-FLIP and XIAP expression in OCI AML2 cells (Figure 7D) lending further support to the importance of NF-κB inhibition in bortezomib-induced sensitization to TRAIL.

### 2.7. Inhibition of NF-κB Sensitized Myelomonocytic Primary AML Cells to TRAIL-Induced Apoptosis

**Figure 8.**
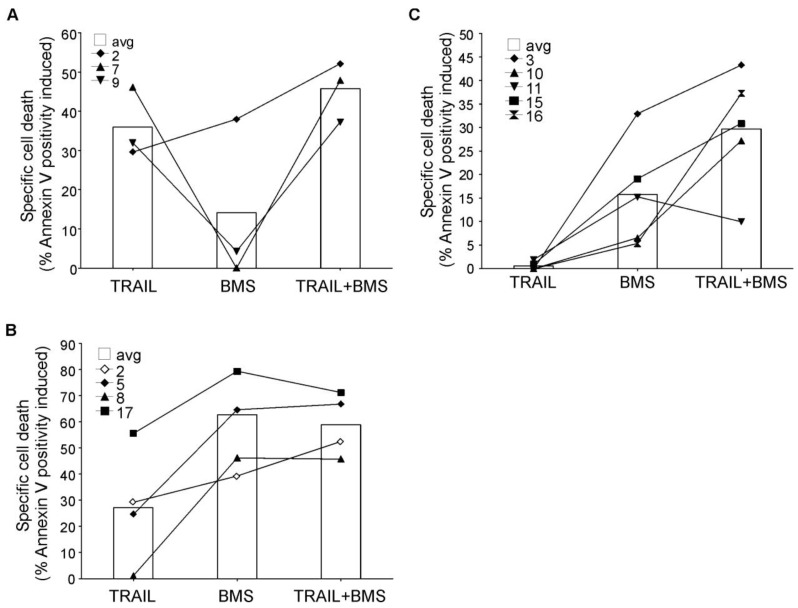
NF-κB inhibition sensitizes resistant primary AML blasts to rhTRAIL. AML blasts were treated with 250 ng/mL of rhTRAIL in the presence or absence of the IKK inhibitor BMS-345541 for 24 h. Induction of cell death was detected with Annexin V staining. The bars indicate the mean values of the samples represented by the individual lines. (**A**) Patient subset with rhTRAIL sensitive AML blasts; (**B**) Patient samples with high BMS-345541 sensitivity; (**C**) rhTRAIL-resistant AML sample group.

Analysis of primary AML blasts further supported the importance of NF-κB activity in rhTRAIL resistance (Figure 8). AML blasts were isolated from bone marrow or peripheral blood of patients and treated with BMS-345541 in combination with rhTRAIL for 24 h. The patient information and the expression profile of the four membrane bound TRAIL receptors (DR4, DR5, Decoy receptor 1 (DcR1 and DcR2) on the cell surface are summarized in Table 2 and Table 3, respectively. We found that a small subset of patient AML cells was already sensitive to TRAIL (patients 2, 7, 9) and in these samples inhibition of NF-κB only marginally increased TRAIL sensitivity (Figure 8A). Some AML samples required NF-κB activity for survival, and accordingly, in these samples administration of BMS-345541 alone already induced high level of cell death (Figure 8B). However, approximately half of the samples tested showed TRAIL resistance and in these samples, inhibition of NF-κB significantly increased TRAIL sensitivity (Figure 8C) indicated by the median difference between the cytotoxic effect of BMS-345541 *vs.* TRAIL+ BMS-345541 and TRAIL *vs.* TRAIL+BMS-345541 (13.94% and 28.02%, respectively), and *p* values determined by two tailed paired student t-test (0.038 and 0.002, for the same treatment pairs).

### 2.8. Discussion

TRAIL targets cancer cells whilst largely sparing normal cells and is therefore a very promising therapeutic modality. However, AML is largely resistant to TRAIL-induced apoptosis, due to inhibition or lack of expression of the apoptosis-inducing TRAIL receptors (DR4, DR5) and overexpression of intracellular anti-apoptotic factors, such as c-FLIP [33]. There is a strong rationale for using bortezomib to overcome TRAIL resistance based on the known influence of bortezomib on the proteins regulating the TRAIL apoptotic pathway, however, currently there is limited information on the potential of bortezomib to sensitize AML to rhTRAIL [38]. Our data reveal that bortezomib exerts potent pro-apoptotic effects against AML *in vitro* in the nanomolar range and also sensitizes AML cells of myelomonocytic/monocytic phenotype, which account for 20-30% of AML cases [39], to rhTRAIL, an effect which is at least partly mediated by inhibition of NF-κB and by reducing c-FLIP and XIAP expression, which is consistent with similar findings in other cancer cells [30,40-42]. Proteasome inhibitors have previously been shown to decrease c-FLIP protein expression in murine leukemia cells, renal cancer cells and chronic lymphocytic leukemia (CLL) cells, which was associated with sensitization to rhTRAIL [30,41].

Previous studies have also established that expression of the caspase-9 and caspase-3 inhibitory protein, XIAP, increases with monocytic differentiation and in adult *de novo* AML high XIAP expression correlates with poor prognosis [43]. It has also been shown that similar to c-FLIP, XIAP is also a transcriptional target of NF-κB [36]. Here we demonstrate higher basal expression of XIAP in the FAB M4 TRAIL-resistant cell lines OCI AML2 and OCI AML3 with a clear reduction post treatment with bortezomib. In the TRAIL-sensitive cell line ML-1, however, the opposite effect occurs, which may suggest differences in regulation of XIAP levels in various AML cells and indicates that in AML, XIAP downregulation may contribute to an enhanced response to TRAIL but it is not essential.

Constitutive NF-κB activity has been detected in a number of hematological malignancies, such as Hodgkin's disease (HD), acute lymphoblastic leukemia (ALL) as well as AML [26,44-46]. As c-FLIP and XIAP are well known target genes of NF-κB, we studied whether inhibition of NF-κB was central to the sensitizing effect of bortezomib on TRAIL-induced apoptosis using a chemical inhibitor of IκB kinase, BMS-345541 [37]. Our data demonstrate an enhancement of TRAIL-induced apoptosis in AML cell lines of myelomonocytic differentiation as well as in primary AML blasts of myelomonocytic/monocytic differentiation by inhibition of IKK. This, together with the detected repression of c-FLIP and XIAP expression after inhibition of IKK supports the hypothesis that sensitisation to TRAIL is at least partly due to bortezomib-mediated inhibition of NF-κB activity/activation. Since TRAIL binding to its receptors also activates NF-κB, it is also plausible that proteasome inhibition may enhance TRAIL-mediated apoptosis principally by blocking TRAIL-mediated NF-κB activation, thereby preventing the expression of anti-apoptotic proteins, including c-FLIP and XIAP. Ubiquitylation is involved in at least three steps in the NF-κB activation pathway: degradation of the NF-κB inhibitor IκB, processing of the p100 and p105 NF-κB precursors and activation of the IκB kinase (IKK) through a degradation-independent mechanism [47]. Bortezomib not only inhibits TRAIL-mediated NF-κB activation by blocking the proteasomal degradation of polyubiquitylated (Lys48)-IκB or processing of p100 and p105, but also leads to the depletion of the cellular pool of free-ubiquitin. This prevents the non-degradational, Lys63-type polyubiquitylation of TRAIL receptor adaptor proteins, which is essential for recruitment of IKKs and thus for NF-κB activation [47]. Of note, Baumgartner and colleagues have shown that enhanced NF-κB activity driven by aberrant IKK activity is a characteristic of myelomonocytic FAB M4 and monocytic/monoblastic M5 AML blasts, but not of AML of early or granulocytic differentiation. Furthermore, in M4/M5 AML blasts treatment with the TRAIL homologue death ligand, tumor necrosis factor (TNF) could further increase NF-κB activity which could be blocked by proteasome inhibition with PS-I [45]. In line with these, we found that all BMS345541-sensitive patient samples were of FAB M4/M5 subtype, reinforcing the finding that myelomonocytic/monocytic AML cells depend on NF-κB activity. Additionally, two out of the four primary samples that could be sensitized to TRAIL with inhibition of NF-κB activation were also FAB M4. These findings strongly indicate that there are differentiation associated differences in TRAIL resistance of AML subtypes and the factor responsible for TRAIL resistance in M4/M5 AML is likely to be NF-κB.

M1- and M2-type AML blasts have higher levels and activity of the multidrug receptor (MDR-1) and P-glycoprotein driven efflux of therapeutic drugs [48,49], however bortezomib was able to block the proteasome in all tested cell lines at a very similar efficacy (IC_50_ value was between 1.9 to 5 nM for all cell lines), eliminating this as a reason for the lack of bortezomib-mediated TRAIL-sensitization. Thus in M1/M2 AML's different mechanisms must block TRAIL-death signaling. Identification of these mechanisms is still awaited. Furthermore, we did not detect any correlation between the effect of bortezomib on the three different enzyme activities of the proteasome, except that the two cell lines with the highest basal proteasome activity (KG-1 and MOLM13) were the most sensitive to the cytotoxic effect of bortezomib. It may indicate that these cells depend on the proteasome to survive. These findings are in agreement with the study of Matondo *et al.*, which also found that elevated 20S expression and high proteasome activity correlated with high bortezomib sensitivity in AML cells [50].

With the realisation of the central role of NF-κB in maintaining leukemic cell survival and drug resistance, ongoing studies are examining the potential of NF-κB inhibition in AML therapy using bortezomib. Importantly, not only the AML blast cells, but also the quiescent leukemic stem cell (LSC) population display high NF-κB activity, which is not the case for normal hematopoietic stem cells and inhibition of NF-κB by bortezomib can effectively target this cell population [27]. The proteasome system is the main, non-lysosomal protein degradation system in the cell controlling the cell cycle, gene transcription, cellular adhesion, *etc.* Systemic blockage of such essential cellular functions can result in severe toxicities such as thrombocytopenia, cardiopulmonary- and neurologic toxicities, which limits the maximum dose and the frequency of bortezomib administration to approximately 1.5 mg/m^2^ bi-weekly [51-54]. The *in vivo* half-life time of bortezomib is in the range of 10-18 h [29] and when administered at the highest recommended dose, proteasomal inhibition was the highest 1 h post administration in peripheral blood mononuclear cells with approximately 70% inhibition of baseline levels that was reduced to about 50% at 24 h [55]. The resulting fluctuating proteasome inhibition, even at the maximum tolerated dose, may be insufficient to achieve potent NF-κB inhibition *in vivo* necessary for effective AML eradication. Thus, alternate strategies, capable of targeting NF-κB in a more sustained fashion than bortezomib would be potentially attractive AML therapeutics [56,57].

## 3. Experimental Section

### 3.1. Cell Culture and Treatments

HL-60 (ATCC), ML-1 (kindly received from Dr. Heinz-Peter Nasheur, Biochemistry Department, NUI Galway) and ML-2 cells (German Collection of Microorganisms and Cell cultures, DSMZ) were maintained in RPMI1640 supplemented with 10% foetal bovine serum (FBS), 2 mM glutamine, 50 U/mL penicillin and 50 μg/mL streptomycin. Cells were seeded at 3 × 10^5^ cells/mL one day prior to treatment. Kasumi, MOLM13 and KG-1 cells (kindly received from Dr. Elisabeth Kremmer, Institute for Molecular Immunology, Munchen, Germany) were maintained in RPMI 1640 supplemented with 10% FBS, 2 mM glutamine, 1 mM sodium pyruvate, 1% non-essential amino acids (NEA), 50 U/mL penicillin and 50 μg/mL streptomycin. Cells were seeded at 5 × 10^5^ cells/mL one day prior to treatment. OCI AML2 and OCI AML3 cells (DSMZ) were maintained in Alpha Minimum Essential Medium (MEM) supplemented with 10% FBS, 2 mM glutamine, 1 mM sodium pyruvate, 1% NEA, 50 U/mL penicillin and 50 μg/mL streptomycin. Cells were seeded at 5 × 10^5^ cells/mL one day prior to treatment.

Bortezomib (Janssen, UK) was dissolved in DMSO and used in a concentration range of 2–100 nM. BMS-345541 (Calbiochem) was dissolved in DMSO and used in a concentration range of 2–10 μM. The formulation of recombinant human TRAIL was non-tagged, fragment 114–281, kindly provided by Wim Quax, University of Groningen, The Netherlands. All materials were from Sigma, unless otherwise stated.

### 3.2. MTT Cell Viability Assay

Cell viability was measured by adding 200 μg/mL MTT (3-(4,5-dimethylthiazol-2-yl)-2,5-diphenyl-tetrazolium bromide) to control and treated cells, and incubated for 3 h at 37 °C. The reaction was stopped and the purple formazan precipitate formed was dissolved in 20% SDS in 50% dimethyl formamide and the color intensity was measured at 550 nm using a Wallac multilabel counter (PerkinElmer). The control value corresponding to untreated cells was taken as 100% and the viability of treated samples was expressed as a percentage of the control.

### 3.3. Annexin V staining

Externalization of phosphatidylserine (PS) on the plasma membrane of apoptotic cells was detected using Annexin V-FITC (IQ Corporation). Briefly, cells were collected by trypsinization and allowed to recover for 10 min in growth medium. The cells were then washed in ice-cold calcium buffer (10 mM HEPES/NaOH, pH 7.4, 140 mM NaCl, 2.5 mM CaCl_2_) and incubated with Annexin V-FITC for 15 min in the dark.

### 3.4. Western Blotting

After treatments cells were lysed in 100 μL buffer (20 mM HEPES, pH 7.5, 350 mM NaCl, 1 mM MgCl_2_, 0.5 mM ethylenediamine-tetraacetic acid (EDTA), 0.1 mM ethylene glycol bis(2-aminoethyl ether)-N,N,N′N′-tetraacetic acid (EGTA), 1% Igepal-630, 0.5 mM dithiothreitol (DTT), 100 μM phenylmethyl-sulphonyl fluoride (PMSF), 2 μg/mL pepstatin A, 25 μM N-Acetyl-Leu-Leu-Nle-CHO (ALLN), 2.5 μg/mL aprotinin and 10 μM leupeptin) for 15 min on ice. Protein concentration was measured using the Bradford method. Protein samples were denatured in Laemmli's sample buffer and boiled for 5 min. Proteins were separated by 10% SDS-PAGE and transferred onto nitrocellulose membrane. Membranes were blocked for 1 h in PBS containing 0.05% Tween 20 and 5% (w/v) non-fat dried milk. The membranes were then incubated for 1 h at room temperature with antibodies to actin (1:500; Sigma) or overnight at 4 °C with antibodies to c-FLIP (1:500; Alexis), or XIAP (1:5000; Assay Design). This was followed by 2 h incubation at room temperature with appropriate secondary antibodies (1:10,000 Thermo Scientific). Protein bands were visualized using Supersignal Ultra Chemiluminescent Substrate (Thermo Scientific) on X-ray film (Agfa).

### 3.5. Proteasome Activity Assay

The chymotryptic, tryptic and PGPH (peptidylglutamyl peptide hydrolyzing) activities of the proteasome were measured by using specific, fluorescently tagged peptide substrates (N-Succinyl-LLVY-AMC, Z-ARR-AMC and Z-LLE-AMC (all from Merck), respectively). Following treatment, cells were lysed in 50 μL of lysis buffer (13 mM Tris buffer containing 5 mM MgCl_2_, pH 7.8) through two freeze-thaw cycles. The samples were divided into 2 × 25 μL and a 175 mL of substrate buffer added (5 mM ATP, 0.5 mM DTT, 5 mM EDTA and 50 μM proteasomal substrate in lysis buffer). Activity was measured as a rate of cleavage of fluorogenic substrates and was determined by monitoring the fluorescence of released AMC (Amido 4-Methylcoumarin), using a fluorescent plate reader (Wallac/Victor 3, PerkinElmer) at an excitation wavelength of 395 ± 25 nm and emission wavelength of 460 ± 40 nm. The assay was run over a period of 35 min (30 cycles, 1 measurement per min) at 37 °C and results were expressed in nmol AMC released/min/mg protein. The protein concentration of the lysates was determined by BCA protein assay (Pierce) according to the manufacturer's instructions.

### 3.6. Isolation and Culture of Primary AML Blasts

BM MNCs were isolated by density gradient centrifugation and cryopreserved in 10% dimethyl sulfoxide (DMSO) freezing medium. Following a rapid thawing and centrifugation to remove DMSO of the freezing medium, cells were cultured in RPMI/10% fetal bovine serum. Cell viability was assessed at the time of thawing the cells and experiments were only carried out in samples that showed at least 75% viability. In addition, samples that showed high rate of spontaneous death and the percentage of live cells reduced to 25% or less at time of harvesting were also excluded from further analysis. The cells were treated with 250 ng/mL of WT rhTRAIL alone for 24 h or in combination with 5 μM BMS-345541 added simultaneously.

### 3.7. Determination of Surface TRAIL Receptor Expression

Cells were removed from culture dishes, harvested by centrifugation and washed twice with 1% BSA in PBS. Cells were incubated with 1:100 dilution of primary antibodies (DR4 and DR5: neutralizing mouse monoclonal antibodies, Alexis, DcR1 and DcR2: neutralizing goat polyclonal antibodies, R&D Systems) in 1% BSA in PBS for 40 min on ice. After two washes in 1% BSA/PBS, cells were resuspended in 1:50 dilution of FITC-labeled secondary antibody and incubated for 40 min on ice. Excess secondary antibody was removed by washing first in 1% BSA in PBS and then PBS. Cells were fixed in 1% formaldehyde/PBS before analysis by flow cytometry (FacsCalibur, Beckton Dickinson).

## 4. Conclusions

In summary, our results provide evidence to justify further investigation into the combination of rhTRAIL with bortezomib or ideally with selective inhibitors of NF-κB, to avoid affecting other cellular processes regulated by the proteasome. Such an approach will potentially lead to more effective AML therapy.

## Figures and Tables

**Table 1.** CI values of synergistic drug interactions in M4/M5 AML cell lines.

**ML-1**	**TRAIL (ng/mL)**	**Bz (nM)**	**CI**
	10.0	7.0	0.93172
	25.0	7.0	0.62343
	50.0	7.0	0.51661
	100.0	7.0	0.69512
	250.0	7.0	1.37954
**ML-2**	**TRAIL (ng/mL)**	**Bz (nM)**	**CI**
	10.0	4.0	0.12802
	25.0	4.0	0.07240
	50.0	4.0	0.09342
	100.0	4.0	0.18088
	250.0	4.0	0.47115
**MOLM-13**	**TRAIL (ng/mL)**	**Bz (nM)**	**CI**
	10.0	8.0	0.96874
	25.0	8.0	0.85746
	50.0	8.0	0.44369
	100.0	8.0	0.48190
	250.0	8.0	0.55473
**OCI-AML2**	**TRAIL (ng/mL)**	**Bz (nM)**	**CI**
	10.0	8.0	1.27244
	25.0	8.0	1.00502
	50.0	8.0	0.62569
	100.0	8.0	0.50518
	250.0	8.0	0.41878
**OCI-AML3**	**TRAIL (ng/mL)**	**Bz (nM)**	**CI**
	10.0	10.0	0.46307
	25.0	10.0	0.40560
	50.0	10.0	0.40126
	100.0	10.0	0.43864
	250.0	10.0	0.42473

**Table 2.** Clinical parameters for patient samples.

**Sample number**	**FAB**	**Sex**	**Age**	**Primary / Secondary?**	**WBC**	**Karyotype**	**Cytogenetic risk group**	**Treatment Intensity**	**Clinical Outcome**
2	M4e	M	51	Primary	91.8	50,XY,+6,+8,+13, inv(16)(pl3q22),+ 22[10]	Favourable	intensive	CR
3	M4	M	33	Primary	71	46,XY,inv(16)(p1 3q22)[10]	Favourable	intensive	CR
5	M4e	M	42	Secondary	107.4	46,XY,inv(16)(pl3q22)[10]	Favourable	intensive	CR
7	unknown	M	81	Primary	40	46,XY [20]	Intermediate	non-intensive	Induction death
8	M4	F	69	Primary	73.5	45,XX,-7[13]	Adverse	non-intensive	Resistant disease
9	unknown	M	85	Primary	45.8	46,XY [20]	Intermediate	non-intensive	CR
10	unknown	F	80	Secondary	89.9			non-intensive	Induction death
11	M1	M	77	Primary	130	46,XY [20]	Intermediate	non-intensive	CR
15	M4	F	72	Relapse	104	46,XX [20]	Intermediate	non-intensive	Resistant disease
16	M1	M	48	Primary	171.5	46,XY [20] FLT3ITD positive	Intermediate	intensive	Resistant disease
17	M5a	M	62	Primary	69.5	46,XY,t(9;11)(p21;q23)[10] ---	Intermediate	intensive	.

WBC: white blood cell count, AMML: acute myelomonocytic leukemia, CR: complete remission, Res Dis: residual disease

**Table 3.** TRAIL receptor cell surface expression in primary AML blasts.

	**DR4**	**DR5**	**DcR1**	**DcR2**
**2**	+	+	-	+++
**3**	+	+	+	-
**5**	+	+	-	+
**7**	-	+++	++	+++
**8**	+	+	+	+
**9**	+	++	-	++
**10**	-	-	-	+
**11**	+	+	-	+
**15**	+	-	++	++
**16**	-	+	+	-
**17**	-	-	-	-

+: low level of expression (geometric mean on histogram is 120-200% of isotype control); ++: medium level of expression (geometric mean is 200–300% of isotype control); +++: high level of expression (geometric mean is more than 300% of isotype control).
